# Geographic characterization of intra-articular pathologies in early-stage avascular necrosis of the hip

**DOI:** 10.1186/s12891-026-09564-9

**Published:** 2026-02-06

**Authors:** Kory B. Dylan Pasko, Hunter Bohlen, Michael S. Kim, Lucas Ray, Brian Gallagher, Anderanik Tomasian, Ryan Fader, Dean Wang

**Affiliations:** 1 Department of Orthopaedic Surgery, University of California Irvine, 101 The City Drive South Pavilion 3 Orange, Irvine, CA 92868 USA; 2 Department of Biomedical Engineering, University of California Irvine, 402 E Peltason Dr, Irvine, CA USA; 3 Georgetown University School of Medicine, 3900 Reservoir Rd NW, Washington, DC USA; 4 Department of Orthopaedic Surgery, University of Minnesota , 500 SE Harvard St, Minneapolis, MN USA; 5 Department of Radiological Sciences, University of California, 101 The City Drive South Orange, Irvine, CA USA; 6 Allina Health Orthopedics, 2805 Campus Drive Suite 465, Plymouth, MN USA

**Keywords:** Avascular necrosis, Osteonecrosis, Core decompression, Hip arthroscopy

## Abstract

**Background:**

Avascular necrosis (AVN) of the hip is a progressive condition that often leads to femoral head collapse, necessitating total hip arthroplasty. While core decompression (CD) is commonly performed for the treatment of early-stage AVN, the incidence and geographic location of concomitant intra-articular pathologies that may contribute to symptoms and disease progression are incompletely understood.

**Hypothesis / Purpose:**

The purpose of this study was to describe the prevalence, anatomical distribution, and diagnostic concordance of MRI vs. arthroscopy in detecting intra-articular pathology in early-stage AVN. We hypothesized that early-stage hip AVN would be associated with a high prevalence of intra-articular pathology in specific anatomic zones, and that higher Ficat stage would be associated with a greater frequency of these lesions.

**Methods:**

Patients who underwent arthroscopic-assisted core decompression (CD) by two surgeons for symptomatic early-stage AVN between March 2020 and December 2024 were retrospectively identified. Preoperative radiographs and MRIs were reviewed by a fellowship-trained musculoskeletal radiologist to characterize AVN involvement of the femoral head, labral tears, chondral pathologies, and synovitis/effusion, using the geographic zone method. MRI interpretations were compared to intraoperative arthroscopic findings. Associations between the Ficat stage and the prevalence of intra-articular pathologies were assessed.

**Results:**

A total of 36 hips in 30 patients were analyzed. Among all hips analyzed, three (8.3%) were Ficat stage 1, 25 (69.4%) were Ficat stage 2a, and eight (22.2%) were Ficat stage 2b. AVN most affected zones 2 and 3 of the femoral head, which frequently corresponded to the locations of labral tears and acetabular transition zone chondral lesions. A high prevalence of labral tears (88.9%), acetabular cartilage lesions (75%), and synovitis (61.1%) was observed. MRI interpretations almost always correlated with arthroscopic findings, although arthroscopy was more sensitive in detecting grade 1 softening of the femoral head and acetabular cartilage. Higher Ficat stage was associated with a higher prevalence of labral tears (p=0.02), acetabular cartilage lesions (p<0.01), femoral head cartilage lesions (p<0.01), and synovitis/effusion (p=0.02).

**Conclusions:**

Early-stage hip AVN is associated with a high prevalence of intra-articular pathologies, with increasing Ficat stage correlating with more frequent labral tears, transition zone and femoral head cartilage lesions, and synovitis/effusion. While MRI accurately identified and localized most intra-articular pathologies, arthroscopy was more sensitive for detecting grade 1 cartilage lesions.

**Clinical Relevance:**

Arthroscopic-assisted core decompression offers an opportunity to identify and treat intra-articular pathology that may contribute to pain and disease progression in early-stage hip AVN. Recognizing the high prevalence and location of these lesions may help optimize surgical decision-making and long-term outcomes.

**Study Design:**

Retrospective case series; Level of evidence, 4.

## What is known about the subject

Core decompression is a widely used joint-preserving treatment for early-stage hip AVN. However, intra-articular pathologies such as labral tears and cartilage lesions are often underdiagnosed and not routinely addressed during treatment. 

## What this study adds to existing knowledge

This study highlights the high prevalence and consistent anatomical distribution of intra-articular lesions in early-stage hip AVN and demonstrates a correlation between increasing disease severity and the frequency of these findings. It also supports the added diagnostic value of arthroscopy over MRI in detecting subtle chondrolabral pathology.

## Introduction

Avascular necrosis (AVN) of the hip is a debilitating condition characterized by compromised vascular supply to the femoral head and neck, leading to bone necrosis and subsequent collapse of the subchondral bone and articular surface [10, 18]. Approximately 5–12% of the 766,000 total hip arthroplasties (THA) are performed annually in the United States to treat hip AVN [3, 7, 23, 26]. Repair and complete healing of pre-collapse AVN of the hip is rare, with 67% of asymptomatic patients and 85% of symptomatic patients progressing from pre-collapse (Ficat I and II) to collapse (Ficat stage III and IV) [17]. While THA has been successful in treating AVN with collapse, the outcomes are worse than in patients with osteoarthritis (OA) [2, 28, 30]. This may be attributed to differences in bone structure and quality in patients with AVN, increasing the surgical complexity at the time of THA. In carefully selected younger patients, hip resurfacing arthroplasty may also represent a viable alternative to THA [31]. Therefore, the early identification of symptomatic early-stage AVN offers a critical window for interventions aimed at preserving the native hip joint.

Historically, isolated core decompression (CD) has been performed for the treatment of pre-collapse hip AVN to relieve intraosseous pressure and promote revascularization of the diseased cancellous bone [1]. Recent studies have highlighted the frequent coexistence of intra-articular pathologies, including cam deformities, labral tears, and chondral lesions, which may contribute to symptomatology and disease progression but were often overlooked with conventional CD, which did not assess or treat any intra-articular pathologies [12, 27]. Magnetic resonance imaging (MRI) remains the gold standard for AVN staging as well as the detection of any concurrent intra-articular pathologies. However, its sensitivity for subtle cartilage or labral abnormalities in particular, remains suboptimal, with arthroscopic evaluation of hip AVN often uncovering additional concurrent pathology not visualized or inaccurately localized on imaging alone [9, 13, 19, 27]. Therefore, simultaneous arthroscopy with CD has been explored, as it enables direct assessment of any intra-articular pathology while allowing for concurrent arthroscopic treatment of these pathologies, direct visualization for any subchondral collapse of the femoral head, and confirmation that no intra-articular extravasation of bone graft has occurred, if bone grafting of the femoral head is performed [14]. Arthroscopic-assisted CD for hip AVN has been shown to improve surgical outcomes and postoperative quality of life while simultaneously reducing the lifetime risk of revision surgery [24, 25]. 

Despite growing interest in arthroscopic-assisted CD for early-stage hip AVN, the prevalence and characteristics of concurrent intra-articular pathologies remain unclear. Further characterization of intra-articular pathologies in early-stage hip AVN would help with their concomitant treatment during surgery and aid in the understanding of the disease process. Thus, this study aimed to geographically characterize intra-articular pathologies associated with early-stage hip AVN in a series of patients treated with arthroscopy-assisted CD. In this cohort of patients, preoperative MRI findings were compared with intraoperative arthroscopic observations, and the association of disease severity and the prevalence of intra-articular pathologies were analyzed. The authors hypothesized that there would be a high prevalence of intra-articular pathologies and that increasing Ficat stage would correlate with an increasing incidence of intra-articular pathologies.

## Materials and methods

A retrospective cohort analysis was performed. All patients treated with arthroscopy-assisted CD by the two senior authors, each at separate institutions, for symptomatic early-stage AVN of the hip between March 2020 and December 2024 were identified. Institutional IRB approvals were obtained for this retrospective review. All patients obtained preoperative three-view radiographs (standing anterior-posterior (AP) pelvis, false profile, and Dunn views) and an MRI. Most of the MRIs obtained were non-contrast MRIs, and a minority of MRIs were MR arthrograms. Surgical treatment consisted of hip arthroscopy to address any intra-articular pathologies, followed by CD with autogenous and allogeneic bone grafting, as described in a prior study [14]. In brief, an interportal capsulotomy was performed to address any intra-articular pathology, such as labrum repair. Traction was released prior to the CD. After completion of the CD with bone grafting, traction was then reapplied, and the arthroscope was re-inserted to ensure no intra-articular extravasation of bone graft. Traction was then again released, and the interportal capsulotomy was closed. Peripheral compartment pathologies, such as cam lesions, were not routinely addressed by one of the surgeons due to the increased risk of iatrogenic fracture with CD.

The lateral center edge angle (LCEA), anterior center edge angle (ACEA), Tönnis angle, crossover sign, and alpha angle were measured on the preoperative radiographs. Cam deformities were defined by an alpha angle > 55° measured on the Dunn view. A 55° alpha angle threshold was selected based on previous arthroscopy-assisted AVN studies to ensure sensitivity in detecting subtle cam deformities. Data on the prevalence, severity, and geographic location of any intra-articular pathologies was recorded by the senior authors via a retrospective review of intraoperative arthroscopy photos and operative notes. The geographic localization of these pathologies was documented using the geographic zone method (zones 1–6 on the femoral head and acetabulum) [11]. Acetabular and femoral head cartilage defects were classified using the Outerbridge classification system, and chondrolabral delamination lesions were classified using the acetabular labrum articular disruption (ALAD) classification [4, 21]. Findings concerning the ligamentum teres were assessed as normal, hypertrophic, or torn.

Radiographs and MRIs were reviewed by a fellowship-trained musculoskeletal radiologist to geographically characterize the severity of AVN, the location of AVN of the femoral head, any labral tears, and any acetabular or femoral head chondral pathologies. MRIs were also assessed for the presence of synovitis and joint effusion. Stage of AVN was assessed using the modified Ficat classification system to be consistent with the majority of prior arthroscopy-assisted core decompression studies [8, 15, 29]. Both the senior authors and the musculoskeletal radiologist were blinded to each other’s collected data.

All statistics were performed using GraphPad Prism (Version 10.4.1). Descriptive statistics were presented as mean ± standard deviation (SD), total number (n), or percentage of the total. Associations between the Ficat stage and the incidence of intra-articular pathologies were calculated using the Chi-Squared or Fisher’s Exact test. Inter-rater agreement was calculated using Cohen’s Kappa (κ). Statistical significance was determined as *p* < 0.05.

## Results

A total of 36 hips in 30 patients were included in the study. The mean age of patients was 34.3 years. There were 26 male (86.7%) and four female (13.3%) patients. The underlying etiology of AVN was idiopathic in 13 patients (43.3%), steroid treatment in eight patients (26.7%), alcohol use in seven patients (23.3%), and related to prior trauma or chemotherapy in two patients (6.7%). Baseline patient demographic data are summarized in Table 1.

**Table 1:** Baseline patient demographic data

Patient Characteristics (*n* = 30)	
Age (years) at MRI, mean ± SD	33.3 ± 7.7
Age (years) at time of surgery, mean ± SD	34.3 ± 7.6
Sex male/female, n (%)	26/4(86.7%/13.3%)
Body mass index (kg/m2) ± SD	29.4 ± 5.2
AVN etiology, n (%)	
Idiopathic	13 (43.3%)
Steroid Use	8 (26.7%)
Alcohol	7 (23.3%)
Other (Trauma, Chemotherapy)	2 (6.7%)
Osteonecrosis Laterality, n (%)	
Bilateral	15 (50%)
Right/Left	8/7 (26.7%/23.3%)
Surgical Hip Characteristics (*n* = 36)	
Ficat stage, n (%)	
Stage 1	3 (8.3%)
Stage 2a	25 (69.4%)
Stage 2b	8 (22.2%)

Data are reported as mean ± SD or count (*n*), percent unless otherwise indicated

*AVN* avascular necrosis, *MRI* magnetic resonance imaging

**Fig. 1:**
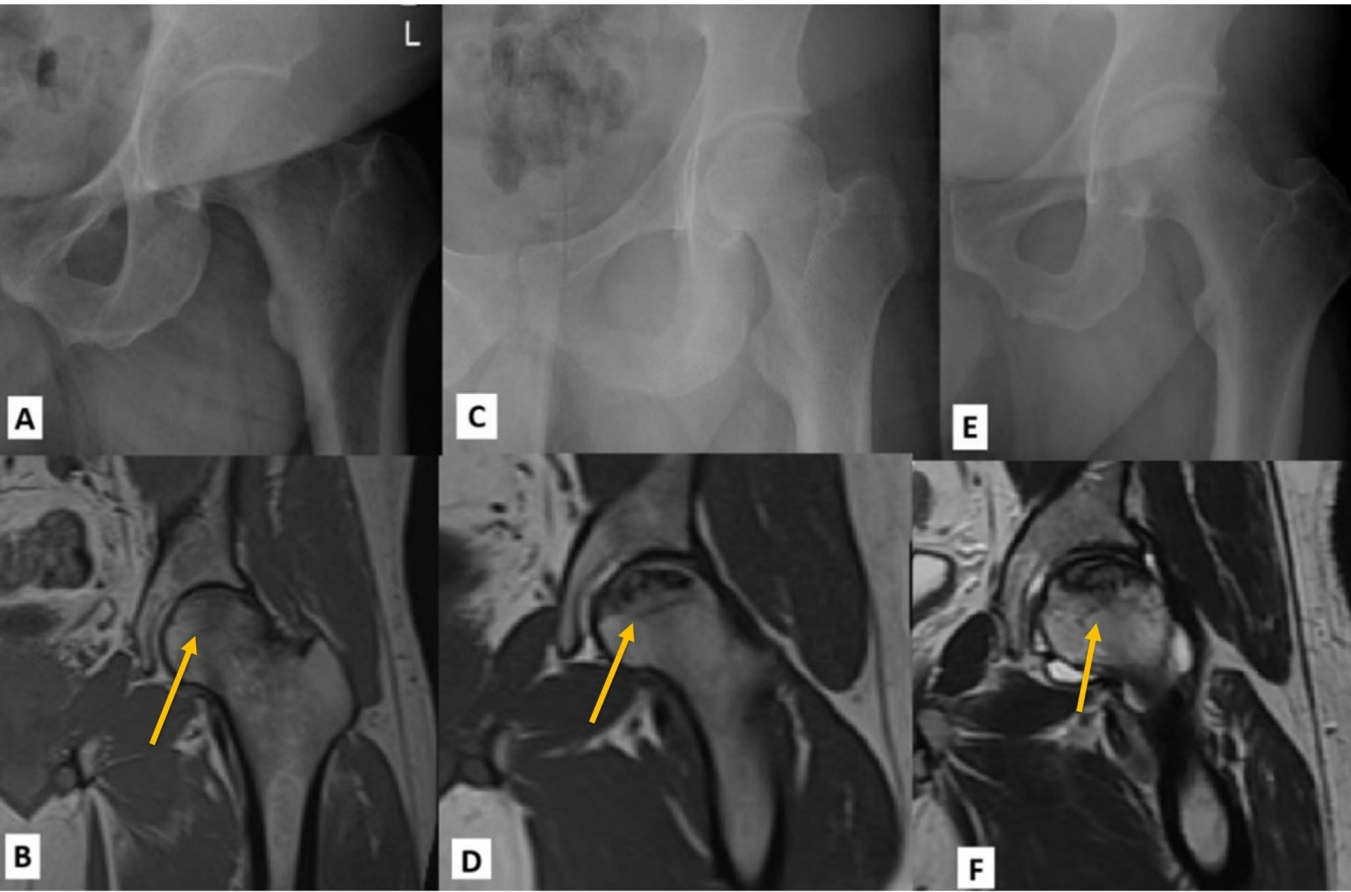
Radiographic progression of AVN of the hip through Ficat stages 1, 2, and 3. (**A**) AP of the left hip of a patient diagnosed with Ficat stage 1 AVN. The plain radiograph appears normal. (**B**) Coronal T2 fat-suppressed MRI of the left hip in the same patient with evidence of Ficat stage 1 AVN of zone 3 of the femoral head characterized by focal marrow edema. (**C**) AP of the left hip of a patient diagnosed with Ficat stage 2a AVN. The plain radiograph shows mild subchondral cysts without any subchondral lucency or cortical collapse. (**D**) Coronal T1 weighted MRI of the left hip in the same patient with evidence of Ficat stage 2a AVN characterized by geographic signal changes in zones 2, 3, and 4 and a sclerotic reactive margin. There is no evidence of cortical collapse. (**E**) AP of the left hip of a patient diagnosed with Ficat stage 2b AVN. The plain radiograph shows a subtle crescent sign. (**F**) Coronal T2 weighted MRI of the left hip in the same patient with evidence of Ficat stage 2b AVN characterized by geographic signal changes in zones 2, 3, and 4 and mild cortical collapse with intact overlying cartilage

Among all hips analyzed, three (8.3%) were Ficat stage 1, 25 (69.4%) were Ficat stage 2a, and eight (22.2%) were Ficat stage 2b (Figure 1). 

**Fig. 2:**
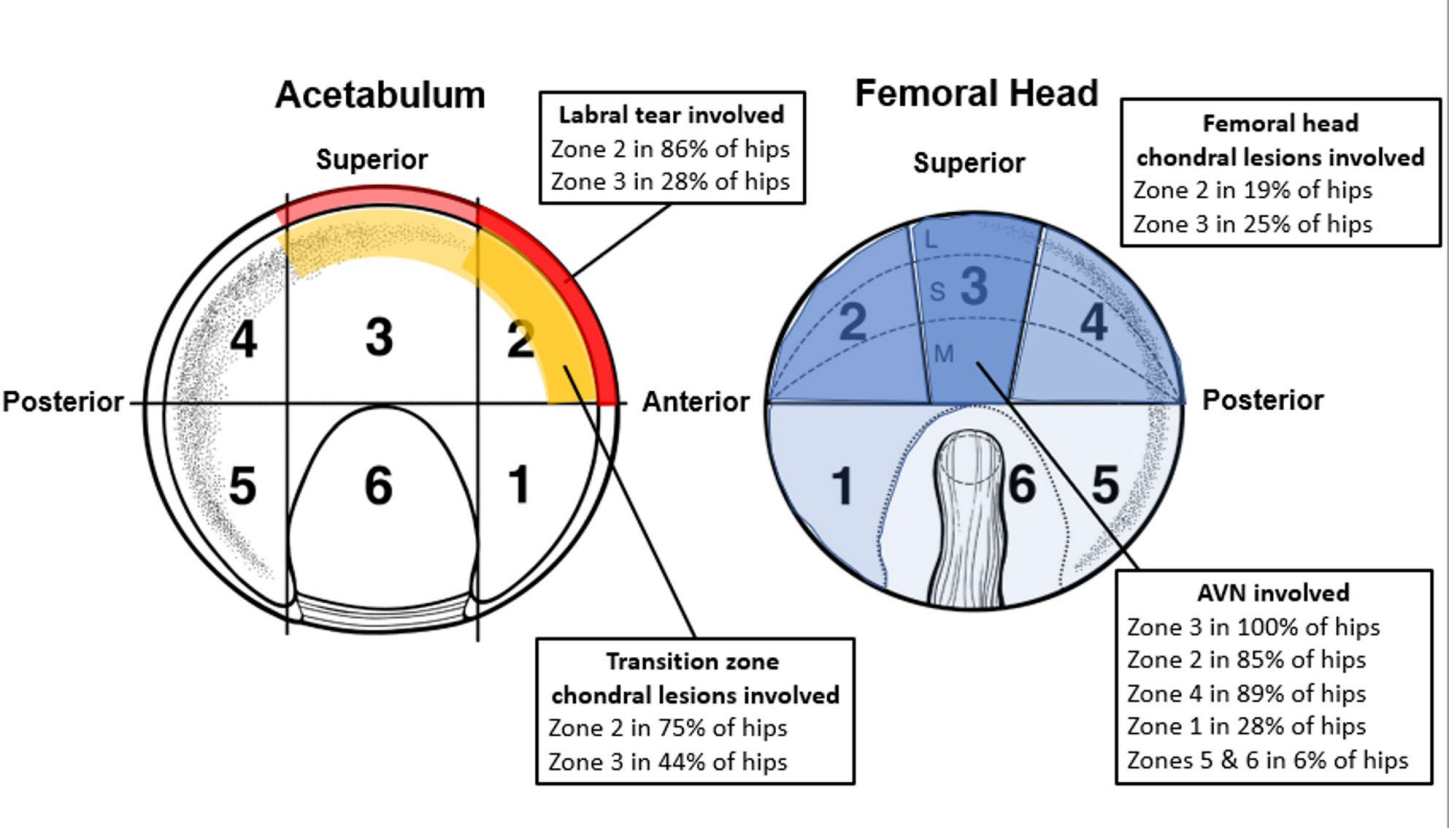
Diagram displaying the geographic zone method for localizing pathology of the acetabulum and femoral head. The frequency of labral tears, transition zone chondral lesions, femoral head chondral lesions, and AVN within each geographic region are reported. Darker colors correspond with a higher frequency of pathologies localized to that geographic zone

AVN most affected zones 2 and 3 of the femoral head, which frequently corresponded to the locations of labral tears and acetabular transition zone chondral lesions (Fig. 2).

**Fig. 3:**
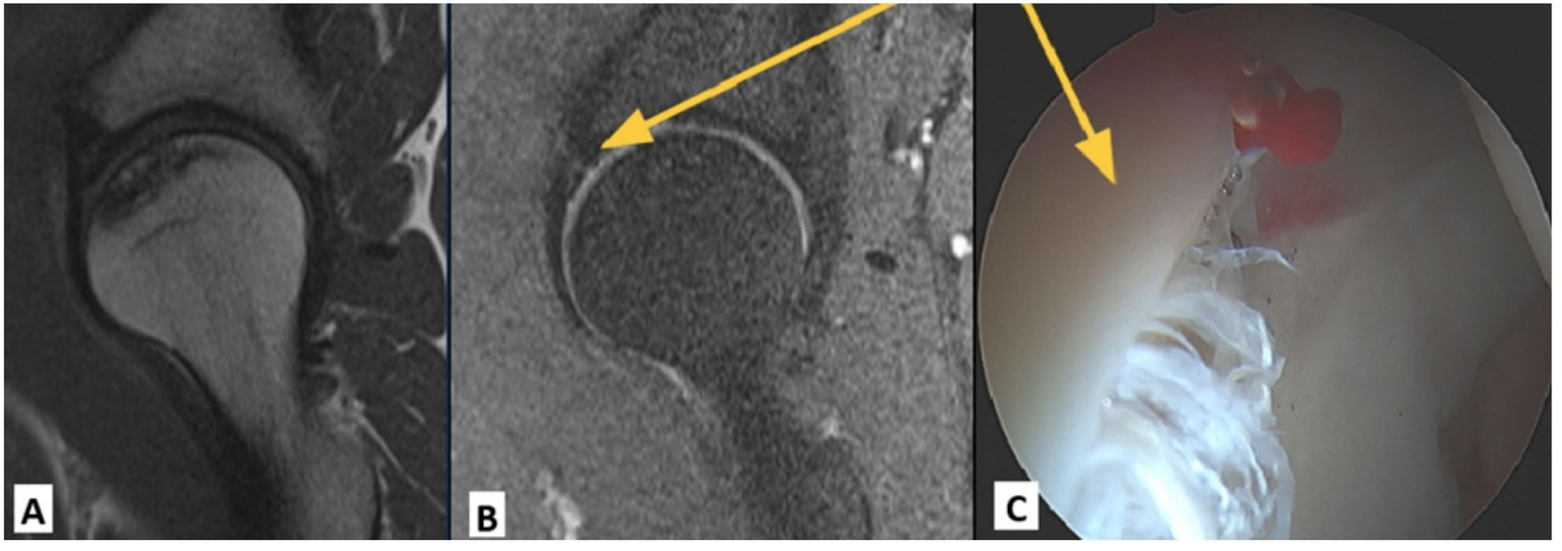
MRI and arthroscopic image of a right hip with Ficat stage 2 avascular necrosis (AVN) of the femoral head. The yellow arrow indicates the location of the labral tear. (**A**) Sagital T1-weighted MRI with crescentic area of Ficat stage 2 AVN of the femoral head characterized by focal bone marrow edema, sclerotic reactive margin, no evidence of subchondral collapse, and preservation of the joint space. The AVN is localized to geographic zones 2 and 3 of the femoral head. (**B**) Sagittal T2-weighted MRI with a zone 2 labral tear. (**C**) Arthroscopic photo viewing form the anterolateral portal redemonstrating the zone 2 labral tear intraoperatively

Arthroscopically, all but one patient in the study population was found to have a concomitant hip pathology. Labral tears were present in 32 (88.9%) hips (Figure 3). 

**Fig. 4:**
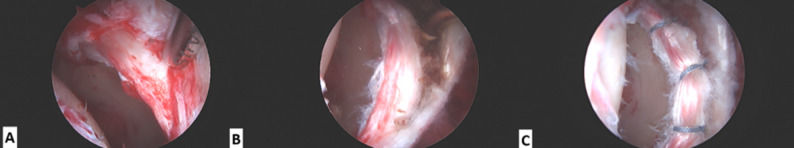
Arthroscopic images of the hip from the anterolateral portal with traction applied demonstrating synovitis with a friable labral tear and subsequent labral repair. (**A**) Significant synovitis with a friable labrum. (**B**) Rim preparation of a torn labrum localized to geographic zones 2 and 3 with delamination from the underlying acetabular rim. (**C**) Postlabral repair with fixation using three suture anchors. There is evidence of associated transition zone chondromalacia

Of these hips, labral tears were present in zone 2 of the acetabulum in 31 (86%) hips and zone 3 in 10 (28%) hips. Labral tears in the setting of hip AVN were often characterized by friable labrum tissue, perhaps due to chronic synovitis, with deterioration of the tissue at the chondrolabral interface (Fig. 4).

**Fig. 5:**
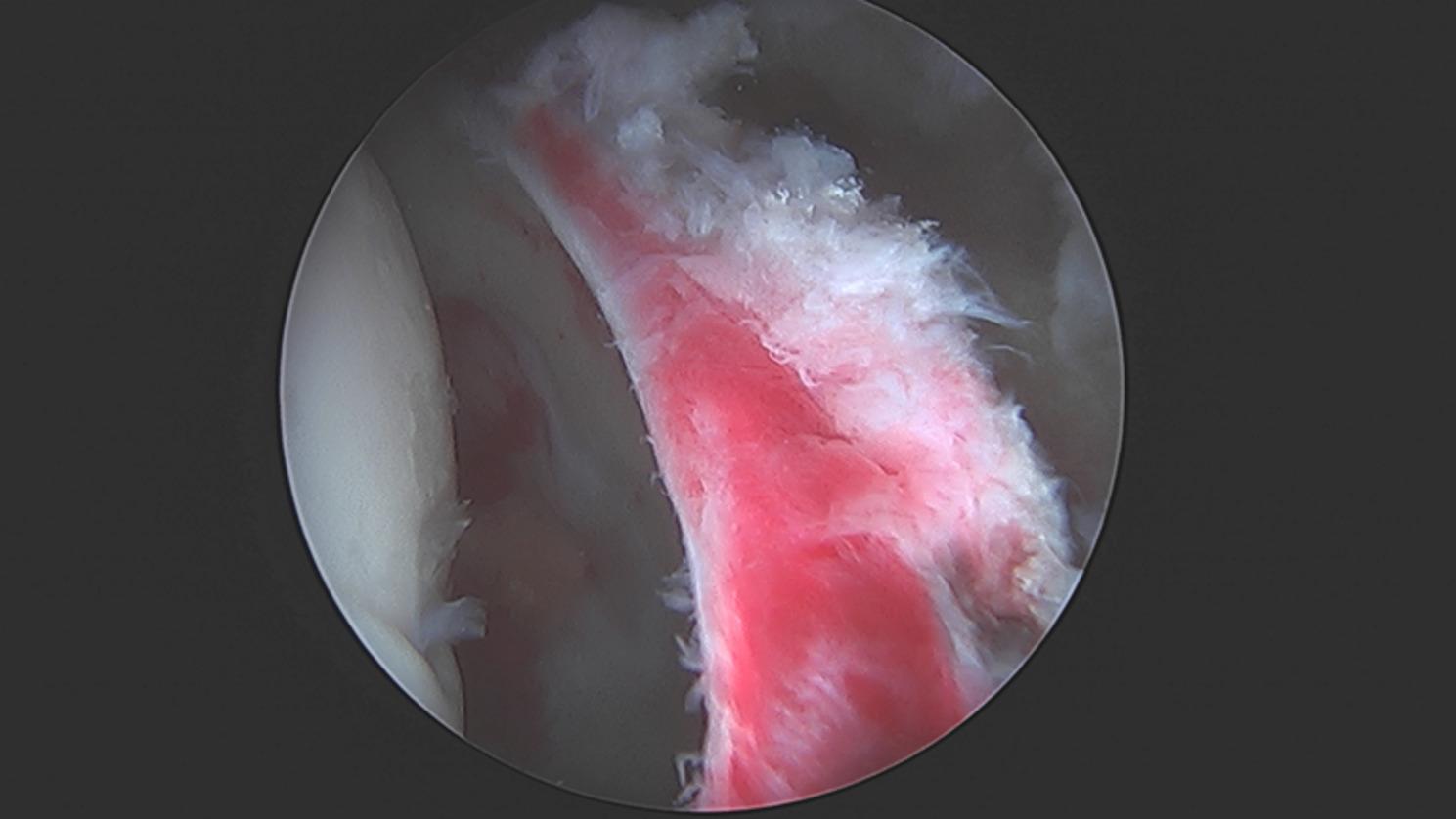
Arthroscopic image of the hip from the anterolateral portal with traction applied demonstrating a zone 2 and 3 labral tear with an associated grade 2 femoral head cartilage lesion characterized by early indentation of the necrotic area of the femoral head

Femoral head cartilage lesions were present in 13 hips (36.1%). Nine (69.2%) were grade 1 softening and four (30.8%) were grade 2 defects observed with early indentation of the necrotic area of the femoral head (Fig. 5).

Acetabular cartilage lesions were present in 27 (75%) hips. Ten (37%) were grade 1, 13 (48.1%) were grade 2, and four (14.8%) were grade 3. All acetabular cartilage lesions were localized in the corresponding geographic zone of necrosis of the femoral head. Synovitis was present in 22 (61.1%) hips and joint effusion was present in 20 (55.6%) hips. Two hips (5.6%) had ligamentum teres hypertrophy and one (2.8%) had partial tearing. The most common concurrent hip arthroscopic procedures performed included synovectomy (94.4%), labral repair (88.9%), subspine and/or acetabular rim decompression (65.5%), and femoroplasty (27.8%). Higher Ficat stage was associated with a higher incidence of labral tears (*p* = 0.02), acetabular transition zone cartilage lesions (*p* < 0.01), femoral head cartilage lesions (*p* < 0.01), and synovitis/effusion (*p* = 0.02). Detailed data of the arthroscopic findings and geographic localization of the concomitant hip pathologies are provided in Table 2.

**Table 2:** Arthroscopic evaluation of accompanying pathologies

Pathology count (*n*), percent (%)		Geographic Location	count (*n*), percent (%)
Labral tear			
Yes	32 (88.9%)	Zone 2 only	22 (61.1%)
No	4 (11.1%)	Zone 3 only	1 (2.8%)
		Zone 2 and 3	9 (25%)
Acetabular cartilage lesions			
Yes	27 (75.0%)	Zone 2 only	11 (40.7%)
Grade 1	10 (37.0%)	Zone 2 and 3	16 (59.3%)
Grade 2	13 (48.2%)		
Grade 3	4 (14.8%)		
No	9 (25.0%)		
Femoral head chondral lesions			
Yes	13 (36.1%)	Zone 2 only	4 (30.8%)
Grade 1	9 (69.2%)	Zone 3 only	6 (46.2%)
Grade 2	4 (30.8%)	Zone 2 and 3	3 (23.0%)
No	23 (63.9%)		
Synovitis			
Yes	22(61.1%)		
No	14 (38.9%)		
Effusion			
Yes	20 (55.6%)		
No	16 (44.4%)		
Ligamentum teres			
Hypertrophy	2 (5.6%)		
Partial tearing	1 (2.8%)		

Data are reported as count (*n*), percent unless otherwise indicated

Cartilage defects graded according to Outerbridge classification system

Geographic location classified by geographic zone method of femoral head and acetabulum

Preoperative radiographs and MRIs were evaluated by a single musculoskeletal fellowship-trained radiologist and compared to intraoperative arthroscopic findings. Preoperative MRI detection of labral tears correlated with arthroscopic findings 100% of the time (κ = 1.00, SE = 0.0, 95% CI [1.00, 1.00]). Comparison of MRI labral tear assessment and arthroscopic findings only differed slightly by geographic localization (κ = 0.873, SE = 0.124, 95% CI [0.630, 1.00], not by detection of presence (κ = 1.0, SE = 0.0, 95% CI [1.0, 1.0]. Preoperative MRI detection of acetabular cartilage lesions correlated with arthroscopic findings in 29 patients (80.6%, κ = 0.533, SE = 0.152, 95% CI [0.235, 0.831]). Preoperative MRI detection of femoral head cartilage lesions correlated with arthroscopic findings in 29 patients (80.6%, κ = 0.533, SE = 0.152, 95% CI [0.235, 0.831]). All 7 of the discrepancies were grade 1 cartilage lesions that were identified intraoperatively but not identified on preoperative MRIs. Detailed data on the preoperative radiographic interpretations and geographic localizations are provided in Table 3.

**Table 3:** Radiographic evaluation of accompanying pathologies

Pathology count (*n*), percent (%)		Geographic Zone	count (*n*), percent (%)
Labral tear			
Yes	32 (88.9%)	Zone 2 only	28 (87.5%)
No	4 (11.1%)	Zone 2 and 3	4 (12.5%)
Acetabular cartilage lesions			
Yes	24 (66.7%)	Zone 2 only	16 (66.7%)
No	12 (33.3%)	Zone 2 and 3	8 (33.3%)
Femoral head cartilage lesions			
Yes	6 (31.0%)	Zone 3 only	1 (16.7%)
No	30 (69.0%)	Zone 2 and 3	3 (50.0%)
		Zone 3 and 4	2 (33.3%)
Cam deformity (ACEA > 55°)			
Yes	24 (66.6%)		
No	12 (33.3%)		
Radiographic Measurement	mean (degrees) ± SD		
LCEA	33.4° ± 6.7		
ACEA	35.5° ± 7.7		
Tonnis angle	3.9° ± 3.7		
Alpha angle	63.3 ± 13.3		
Crossover sign	count (n), percent (%)		
Yes	12 (33.3%)		
No	24 (66.7%)		

Data are reported as mean ± SD or count (*n*), percent unless otherwise indicated

*LCEA* lateral center edge angle, *ACEA* anterior center edge angle

## Discussion

This study highlights the frequent occurrence of concomitant intra-articular hip pathologies in patients with early-stage AVN of the hip and a positive association between Ficat stage and the prevalence of intra-articular pathology. Geographic localization indicated that these pathologies, most frequently labral tears (88.9%) and acetabular transition zone chondral lesions (75.9%), are typically concentrated in geographic zones 2 and 3, corresponding to the areas of femoral head AVN involvement. Additionally, the association between higher Ficat stage and an increased incidence of labral tears, acetabular and femoral head cartilage lesions, synovitis, and joint effusion found in this study highlights the progressive nature of intra-articular damage as the severity of AVN advances.

With regard to the frequent co-occurrence of intra-articular pathology in patients with early-stage AVN, our findings align with prior studies. For example, Serong et al. reported that among 27 patients undergoing arthroscopic-assisted CD for AVN, 85.2% had concurrent labral tears and 74.1% had chondral damage, 12 of which localized to the femoral head above the area of femoral head necrosis [27]. However, their study also highlighted distinct difficulties in using preoperative imaging for the assessment of the labral and chondral status, with preoperative MRI evaluation frequently differing from intraoperative arthroscopic findings. They attributed these discrepancies to several factors, including the resolution of the MRI, variations in imaging protocols, incomplete or inaccurate presumptive diagnosis, and the highly curved and obliquely oriented joint [13, 19, 27]. In our study, MRI evaluation of labrum and chondral surfaces almost always correlated with arthroscopic findings, aside from the detection of grade 1 softening of the femoral head cartilage. These findings highlight the ability of modern MRI systems with optimized hip sequences, in conjunction with the expertise of a fellowship-trained musculoskeletal radiologist, to overcome previous shortfalls with traditional low-field MRI to accurately evaluate concurrent intra-articular hip pathologies.

The high frequency of geographic localization of AVN lesions to zones 2 and 3 of the femoral head found in this study may be a reflection of the higher biomechanical stresses experienced in these regions. Subsequently, increasing structural instability of the subchondral bone leading to overlying cartilage softening, superficial fissuring, lift-off, and breakdown over the course of disease progression likely explains the 100% correlation between the geographic location of femoral head cartilage defects and AVN seen in this study [16]. Similarly, the location of acetabular cartilage defects was found to always correlate with the location of femoral head necrosis in this study. These findings correspond with prior imaging studies that described a predilection for concomitant hip pathologies seen in AVN and femoroacetabular impingement to the weight-bearing regions of the acetabulum, leading to focal cartilage breakdown and labral dysfunction [19, 27]. While this theory explains the frequent overlap between the geographic localization of AVN and intra-articular pathology, our study also found a higher prevalence of labral tears (88.9%) and acetabular chondral lesions (75.9%) than femoral head chondral lesions (31%), which had not been previously described. These findings highlight the multifactorial and incompletely understood nature of AVN progression and its interaction with surrounding hip joint structures. Given recent work suggesting that subchondral insufficiency fractures of the knee (SIFK) may develop secondary to meniscal pathology, it is conceivable that idiopathic hip AVN in some cases could represent a similar mechanism driven by labral dysfunction and altered load distribution [5, 6, 20, 32]. Labral tears may induce focal stress concentration and subchondral compromise, potentially contributing to ischemic injury in susceptible individuals. Further research is needed to investigate the role of these intra-articular pathologies in the pathogenesis and disease progression of hip AVN.

Furthermore, our study revealed a high incidence of synovitis (61.1%) and joint effusion (55.6%) in early-stage AVN and is the first to describe an association between a higher Ficat score and the incidence of synovitis and joint effusion. Prior studies by Rabquer et al. have suggested that synovial inflammation may play a key role in the disease progression of AVN, in addition to compromised blood supply, via inflammatory mediators that exacerbate bone resorption and joint degradation [22]. Similar to our results, they found significant synovial inflammation in 65% of patients at the time of THA for AVN, 35% of which had no known inflammatory disease comorbidity [22]. The inflamed synovium in AVN was found to consist primarily of CD68 + macrophage and CD4 + T-cells, which differed from the composition seen in inflammatory arthropathies such as rheumatoid arthritis. These findings, combined with the findings of our current study, underscore the need for further research to elucidate the incompletely understood role of synovial inflammation in the pathogenesis and disease progression of AVN of the hip.

## Limitations

There are several limitations to this study. This was a retrospective study of two surgeons’ cohorts, which limits the generalizability of this study. Second, the relatively small sample size reduced the statistical power for subgroup analyses and limited in-depth statistical analysis. Future prospective studies with a larger cohort are needed to increase the statistical power and verify the external validity of these findings. Another limitation is the lack of histological confirmation of osteonecrosis. Histological evaluation was not feasible in this study, as bone tissue was not sampled during arthroscopy-assisted core decompression. Future studies incorporating histological confirmation are needed to further differentiate true osteonecrosis from subchondral insufficiency fractures. Lastly, our study did not evaluate the long-term outcomes of arthroscopic-assisted CD. Further research is needed to fully understand if combined CD and arthroscopy improve the overall outcomes achieved by CD alone.

## Conclusions

This study found a high prevalence of concurrent intra-articular pathologies in patients with early-stage hip AVN, with a higher Ficat stage associated with a higher prevalence of labral tears, transition zone and femoral head cartilage lesions, and synovitis/effusion. The geographic localization of these pathologies often correlates with the regions of AVN involvement. While MRI accurately identified and localized most intra-articular pathologies, arthroscopy was more sensitive for detecting grade 1 cartilage lesions.

## Data Availability

The datasets used and/or analysed during the current study are available from the corresponding author on reasonable request.
